# Polarization‐ and Frequency‐Selective Light Amplification in Magneto‐Optic Photonic Time Crystals

**DOI:** 10.1002/nap2.70069

**Published:** 2026-04-14

**Authors:** Shuqiao Xu, Haoshuang Zhong, Zicheng Yang, Guoyong Zhang, Hao Chen, Xiao Chen, Bo Chu, Zhuo Chen, Zhenlin Wang

**Affiliations:** ^1^ School of Physics and National Laboratory of Solid State Microstructures Nanjing University Nanjing China

**Keywords:** light amplification, magneto‐optic photonic time crystal, polarization and frequency control

## Abstract

Light amplification in photonic time crystals (PTCs), enabled by their momentum bandgap, is a well‐established phenomenon. However, prior research has predominantly focused on achieving a substantial gap, whereas the controlled manipulation of light properties remains largely unexplored. Here, we exploit spatially finite magneto‐optic photonic time crystals (MO‐PTCs) to achieve polarization‐ and frequency‐selective light amplification. By magnetizing the MO‐PTC, we dynamically tune the effective permittivity of extraordinary light (e‐light), thereby controlling its amplification threshold. Applying periodic modulation to the diagonal elements of the dielectric tensor enables selective amplification of either ordinary light (o‐light) or extraordinary light, depending on which has the lower threshold. Furthermore, modulating the off‐diagonal elements induces an effective modulation comprising both the fundamental frequency and its second harmonic in the extraordinary light. Adjusting the extraordinary light's permittivity allows preferential amplification of either the fundamental or second‐harmonic component based on their respective thresholds. This work advances the understanding of spatially finite PTCs and introduces a novel approach to light manipulation in time‐varying media.

## Graphical abstract

This work pioneers light control in magneto‐optic photonic time crystals via threshold engineering. Magnetic tuning of permittivity enables selective amplification of polarization states and frequency components, including harmonic generation without nonlinear effects. Our finite‐system approach overcomes traditional limitations, offering new possibilities for reconfigurable photonic devices in time‐varying media.

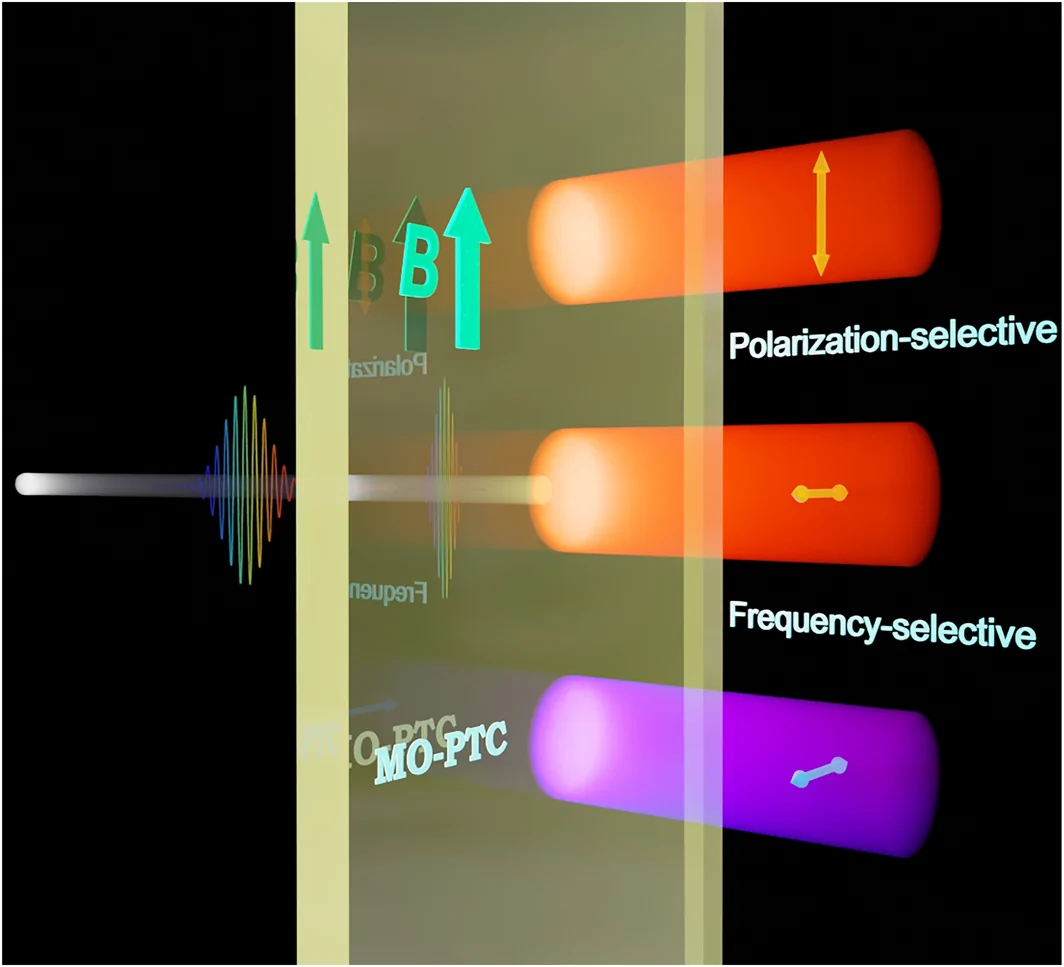

## 1 Introduction

Time‐varying materials have spurred extensive theoretical and applied research in light manipulation owing to their unique temporal modulation capabilities [1, 2, 3]. The foundational concept of a temporal interface—analogous to a spatial interface—was first proposed by F. Morgenthaler in 1958 [4], revealing phenomena such as transmission, reflection, and frequency shifts at temporal boundaries [5, 6, 7]. Subsequent studies by D. Holberg and K. Kunz further explored temporal periodic modulation [8]. Notably, a dielectric‐metal temporal interface with an opposite sign of dielectric constant has been shown to hold and amplify electromagnetic waves [9]. This field has expanded significantly in recent years, with innovative demonstrations including temporal aiming [10], polarization conversion [11], nonreciprocity [12], temporal double‐slit diffraction [13], second‐harmonic generation [14], and optical fission [15].

The momentum bandgap, a key feature of photonic time crystals (PTCs), hosts a pair of eigenmodes with conjugate eigenfrequencies [1, 16, 17, 18]. Under external modulation, energy injection can excite an exponentially growing wave within this bandgap, a phenomenon leveraged in applications such as lasing [19], nonlinear optics [20], and radiation [21, 22]. Similar to spatial moiré lattices, temporal moiré leads to extremely narrow bands and light temporal localization [23, 24]. Conversely, the exponentially decaying eigenmode, which exhibits phase sensitivity, enables controlled absorption [25, 26]. Beyond these dynamical properties, the topological characteristics of PTCs have also garnered significant interest [27, 28]. By integrating spatial and temporal periodic modulations, novel mechanisms for light–matter interaction emerge, including optical isolation [27], luminal metamaterials [28, 29], and mixed momentum‐energy gaps [30, 31, 32].

Although most studies model PTCs as infinite media, realistic systems are inherently spatially finite. In such finite PTC structures (e.g., slabs or spheres), light amplification the modulation depth to exceed a critical threshold [33, 34]. Currently, research on finite‐sized systems has primarily focused on isotropic media, including studies on transmission and reflection [18, 35], and parametric resonance [33, 36], whereas discussions on anisotropic media have not yet been addressed. In this work, we demonstrate a novel mechanism for polarization‐ and frequency‐selective light amplification in spatially finite photonic time crystals (PTCs) using magneto‐optic effects. Through analytical modeling, we reveal that perpendicular magnetization induces anisotropic effective permittivities for ordinary light (o‐light) and extraordinary light (e‐light), creating polarization‐dependent amplification thresholds. By strategically modulating the diagonal elements of the dielectric tensor, we achieve polarization‐selective amplification of the lower‐threshold mode. Furthermore, fundamental frequency modulation of off‐diagonal elements generates both fundamental and second‐harmonic components in e‐light, whose threshold separation enables frequency selection via magnetization control. Finite‐difference time‐domain simulations provide quantitative validation of our theoretical predictions. This work establishes threshold engineering in magneto‐optic PTCs as a powerful approach for spatiotemporal light manipulation, providing some new functions for light amplification.

## 2 Threshold of a PTC Slab

In a finite PTC slab, light amplification occurs within the momentum gap, whereas radiation losses cause dissipation outside the PTC. Remarkably, certain eigenmodes—analogous to self‐excited oscillations—can be sustained when the amplification and dissipation processes achieve equilibrium [33]. As illustrated in Figure 1a, we consider a PTC slab of thickness *d* embedded in a background medium with refractive index *n*_*b*_. Considering the modulation as follows:

(1) $$\varepsilon \left(t\right)=\bar{\varepsilon }\left[1+a\;\cos\left(\Omega t\right)\right],$$

under the weak‐modulation approximation (*a* ≪ 1), these steady‐state modes need to be satisfied:

(2) $$\begin{array}{c} N\;\cos\left(2\pi \sqrt{\bar{\varepsilon }}L\right)\pm \cos\left(\frac{\pi \sqrt{\bar{\varepsilon }}La}{2}\right)=0,\\ N=\frac{n_{b}^{2}-\bar{\varepsilon }}{n_{b}^{2}+\bar{\varepsilon }},\;L=\frac{d\Omega }{4\pi c}, \end{array}$$

where the + and − signs correspond to symmetric and antisymmetric electric field distributions along the *z*‐direction, respectively [33]. Equation (2) establishes a relationship between the dimensionless thickness *L* and the modulation depth *a* that supports stable oscillation of these modes without attenuation (see Supporting Information S1). To further investigate the modulation threshold, we expand the transcendental Equation (2) in the limit of small modulation depth (*a* → 0), yielding the following:

(3) $$\left|a\right|=\frac{2\sqrt{2}}{\pi \sqrt{\bar{\varepsilon }}L}\sqrt{1\pm N\;\cos\left(2\pi \sqrt{\bar{\varepsilon }}L\right)}.$$

**FIGURE 1:**
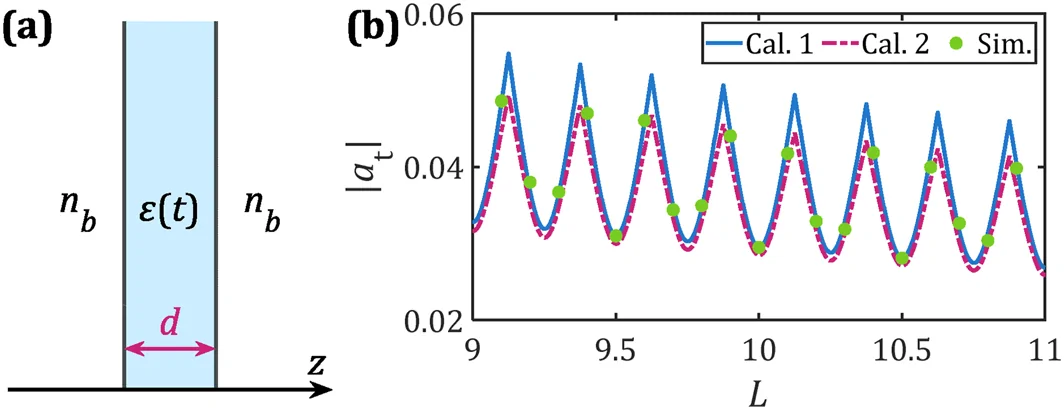
A PTC with finite thickness and its modulation depth threshold. (a) Schematic drawing of a PTC with thickness *d* and background refractive index *n*_*b*_. (b) Modulation depth threshold of a PTC to produce exponentially increasing waves (*n*_*b*_ = 1, $\bar{\varepsilon }$ = 4). The blue solid line and the red dashed line represent the calculation results of Equations (2) and (3), respectively. The green dots indicate the simulation results.

Equation (3) reveals that the modulation depth threshold *a*_*t*_ exhibits oscillatory behavior with an overall decaying envelope as the average optical distance $\left(\sqrt{\bar{\varepsilon }}L\right)$ increases. The minimal threshold condition occurs when:

(4) $$L=\frac{m}{2\sqrt{\bar{\varepsilon }}}\overset{\mathrm{def}}{=}L_{m}^{\min},m=0,1,2,\text{⋯},$$

and the maximal threshold condition occurs when:

(5) $$L=\frac{2m+1}{4\sqrt{\bar{\varepsilon }}}\overset{\mathrm{def}}{=}L_{m}^{\max},m=0,1,2,\text{⋯},$$

Figure 1b presents excellent agreement between theoretical predictions and numerical simulations for the case where *n*_*b*_ = 1 and $\bar{\varepsilon }$ = 4, validating the analytical approach.

## 3 Polarization‐Selective Light Amplification

In this work, we propose a novel and robust principle for polarization manipulation: engineering distinct amplification thresholds for different polarization states. We implement this concept using a magneto‐optic crystal as our polarization‐anisotropic platform. For light propagating along the *z*‐direction under an external magnetic field (*x*‐direction), the relative permittivity tensor takes the form:

(6) $$\varepsilon =\left[\begin{array}{ccc} \varepsilon & 0 & 0\\ 0 & \varepsilon & -i\varepsilon_{m}\\ 0 & i\varepsilon_{m} & \varepsilon \end{array}\right],$$

where we neglect the diagonal element anisotropy for simplicity [37]. Solving Maxwell's equations reveals two orthogonally polarized modes with distinct effective permittivities:

(7) $$\begin{array}{l} o‐\text{light}:\varepsilon_{o}=\varepsilon ,\\ e‐\text{light}:\varepsilon_{e}=\varepsilon -\varepsilon_{m}^{2}/\varepsilon . \end{array}$$

Here, o‐light corresponds to *E*_*x*_ polarization (parallel to the magnetic field), whereas e‐light is dominated by *E*_*y*_ polarization (perpendicular to the field). This inherent polarization‐dependent response enables threshold‐based selective amplification through appropriate modulation schemes.

We first demonstrate polarization‐selective light amplification by modulating the diagonal elements of the dielectric tensor according to Equation (1). By substituting Equation (1) into Equation (7), we obtain the approximate modulation dynamics for e‐light (see Supporting Information S1):

(8) $$\begin{array}{l} \varepsilon_{e}\approx {\bar{\varepsilon }}_{\text{eff}}\left[1+a_{\text{eff}}^{\left(1\right)}\;\cos\left(\Omega t\right)\right],\\ {\bar{\varepsilon }}_{\text{eff}}=\frac{2{\bar{\varepsilon }}^{2}-2\varepsilon_{m}^{2}-a^{2}\varepsilon_{m}^{2}}{2\bar{\varepsilon }},\\ a_{\text{eff}}^{\left(1\right)}=\left({\bar{\varepsilon }}^{2}+\varepsilon_{m}^{2}\right)\frac{a}{{\bar{\varepsilon }}_{\text{eff}}\bar{\varepsilon }}. \end{array}$$

Equation (8) exhibits a form analogous to Equation (1), enabling us to calculate the e‐light threshold ($a_{t}^{e}$) through Equation (2) by substituting the effective average permittivity and modulation depth derived from Equation (8). Notably, the o‐light threshold ($a_{t}^{o}$) remains unaffected by the external magnetic field, whereas the e‐light threshold shows significant magnetic field dependence, as illustrated in Figure 2a. This behavior originates primarily from the magnetic field‐induced reduction in e‐light's effective average permittivity (increasing *ε*_*m*_). Equation (4) shows that this reduction shifts the minimum $a_{t}^{e}$ toward smaller *L* values. Figure 2b quantifies the threshold difference ($\Delta a_{t}=\left|a_{t}^{o}\right|-\left|a_{t}^{e}\right|$), revealing a pronounced maximum at *ε*_*m*_ ≈ 0.63 and vanishing separation near *ε*_*m*_ ≈ 0.9.

**FIGURE 2:**
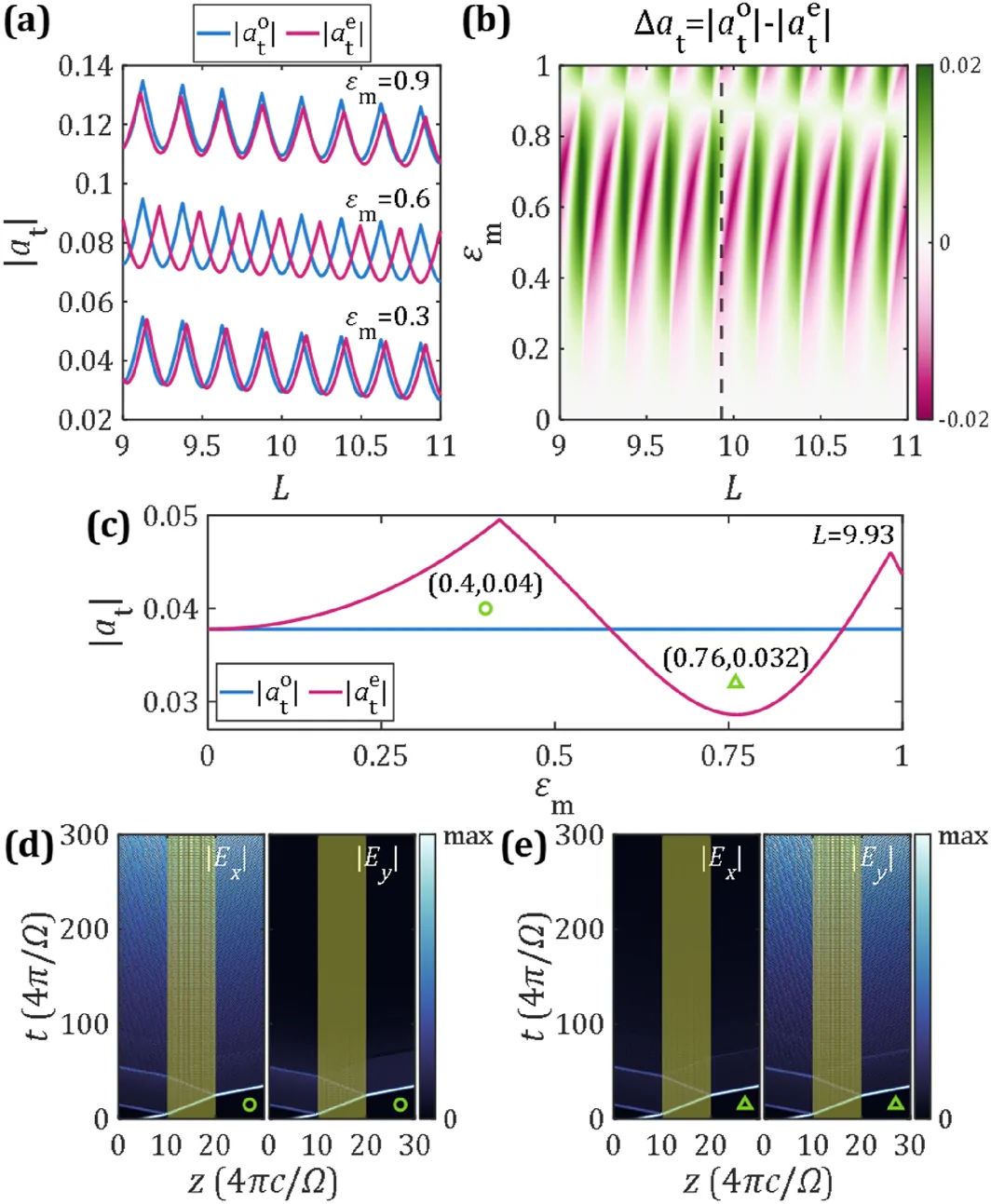
Polarization‐selective light amplification in the MO‐PTC. (a) Threshold modulation depths of ordinary ($a_{t}^{o}$, blue) and extraordinary ($a_{t}^{e}$, red) light at different *ε*_*m*_. Each curve is shifted vertically by 0.04 for clarity. (b) Threshold difference $\Delta a_{t}=\left|a_{t}^{o}\right|-\left|a_{t}^{e}\right|$. (c) Threshold curves at fixed thickness *L* = 9.93, with simulation parameters marked for (i) o‐light amplification (circle, *a* = 0.04, *ε*_*m*_ = 0.4) and (ii) e‐light amplification (triangle, *a* = 0.032, *ε*_*m*_ = 0.76). (d, e) Finite‐difference time‐domain (FDTD) simulation results demonstrating polarization‐dependent amplification. The MO‐PTC slab (centered at *z* = 15, yellow area) interacts with an initially circularly polarized wave packet (centered at *z* = 5, center wavelength = wave packet width = 4π*c*/Ω), showing selective amplification of either *E*_*x*_ (o‐light) or *E*_*y*_ (e‐light) components depending on the applied parameters.

To achieve effective polarization control via external magnetic field tuning, the slab thickness must be selected to ensure that the threshold difference (Δ*a*_t_) can assume both positive and negative values. This requirement excludes thicknesses corresponding to the extremal points ($L_{m}^{\min}$ and $L_{m}^{\max}$) of the threshold curve. As illustrated in Figure 2c, we select *L* = 9.93, with simulation parameters marked by a circle for o‐light amplification (*a* = 0.04, *ε*_*m*_ = 0.4, Δ*a*_t_ < 0) and a triangle for e‐light amplification (*a* = 0.032, *ε*_*m*_ = 0.76, Δ*a*_t_ > 0). Figure 2d,e presents the corresponding evolution of the electric field. The simulation initializes with a circularly polarized wave packet (center wavelength = 4π*c*/Ω, wave packet width = 4π*c*/Ω, broadband source) at *z* = 5, whereas the MO‐PTC slab is centered at *z* = 15. Temporal evolution reveals distinct polarization‐selective amplification (see Video S1): For the circle parameters, *E*_*x*_ (o‐light) amplifies, whereas *E*_*y*_ (e‐light) dissipates (Figure 2d); for the triangle parameters, *E*_*y*_ (e‐light) amplifies, whereas *E*_*x*_ (o‐light) dissipates (Figure 2e). The amplified light field remains at half the modulation frequency. This clear polarization discrimination demonstrates the efficacy of our scheme for magneto‐optically controlled light in finite PTC systems.

## 4 Frequency‐Selective Light Amplification

We now demonstrate frequency‐selective amplification through modulation of the dielectric tensor's off‐diagonal elements:

(9) $$\varepsilon_{m}=\varepsilon_{m0}+a_{m}\;\cos\left(\Omega t\right).$$

As derived from Equation (7), o‐light remains unmodulated, whereas e‐light experiences an effective modulation described by the following equation (see Supporting Information S1):

(10) $$\begin{array}{l} \varepsilon_{e}={\bar{\varepsilon }}_{\text{eff}}\left[1+a_{\text{eff}}^{\left(1\right)}\;\cos\left(\Omega t\right)+a_{\text{eff}}^{\left(2\right)}\;\cos\left(2\Omega t\right)\right],\\ {\bar{\varepsilon }}_{\text{eff}}=\frac{2\varepsilon^{2}-2\varepsilon_{m0}^{2}-a_{m}^{2}}{2\varepsilon },\\ a_{\text{eff}}^{\left(1\right)}=-\frac{2\varepsilon_{m0}a_{m}}{{\bar{\varepsilon }}_{\text{eff}}\varepsilon },\;a_{\text{eff}}^{\left(2\right)}=-\frac{a_{m}^{2}}{2{\bar{\varepsilon }}_{\text{eff}}\varepsilon }. \end{array}$$

Remarkably, although the applied modulation in Equation (9) contains only the fundamental frequency (Ω), the resulting e‐light modulation in Equation (10) exhibits both fundamental and second‐harmonic components. Figure 3a presents the calculated amplification thresholds for both frequency components: the fundamental ($a_{m,t}^{\Omega /2}$) and its second harmonic ($a_{m,t}^{\Omega }$). The fundamental frequency threshold decreases sharply with increasing *ε*_*m*0_, whereas the second‐harmonic threshold shows little change. This contrasting behavior enables frequency selection through the threshold difference ($\Delta a_{m,t}=\left|a_{m,t}^{\Omega /2}\right|-\left|a_{m,t}^{\Omega }\right|$), which undergoes a sign reversal [see Figure 3b]. By tuning the DC magnetic field to control *ε*_*m*0_, we achieve switchable amplification of either the fundamental or second‐harmonic component in the same MO‐PTC structure.

**FIGURE 3:**
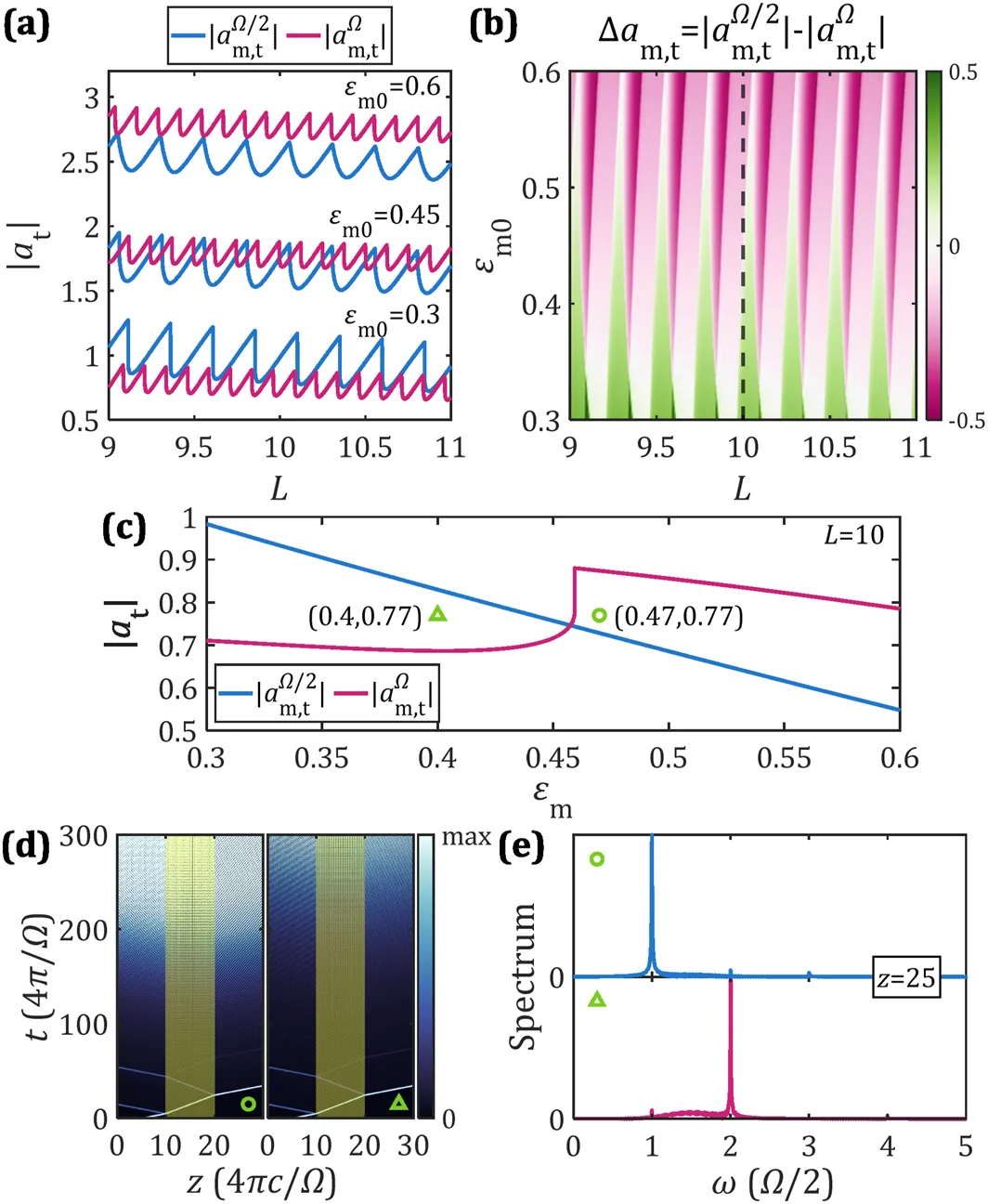
Frequency‐selective light amplification in the MO‐PTC. (a) Threshold modulation depths of the fundamental frequency ($a_{m,t}^{\Omega /2}$, blue) and its second‐harmonic component ($a_{m,t}^{\Omega }$, red) at different *ε*_*m*0_. Each curve is shifted vertically by 1 for clarity. (b) Threshold difference $\Delta a_{m,t}=\left|a_{m,t}^{\Omega /2}\right|-\left|a_{m,t}^{\Omega }\right|$. (c) Threshold curves at fixed thickness *L* = 10, with simulation parameters marked for (i) fundamental frequency amplification (circle, *a*_*m*_ = 0.77, *ε*_*m*0_ = 0.47) and (ii) second‐harmonic amplification (triangle, *a*_*m*_ = 0.77, *ε*_*m*0_ = 0.4). (d) FDTD simulation results showing |*E*_*y*_| field evolution. The MO‐PTC slab (centered at *z* = 15, yellow area) interacts with an initial wave packet (centered at *z* = 5, center wavelength = 8π*c*/3 Ω, wave packet width = 2π*c*/Ω), showing light amplification. (e) Frequency spectrum via fast Fourier transform of *E*_*y*_ at *z* = 25 (*t* = 0–300), clearly resolving the Ω/2 (*ε*_*m*0_ = 0.47) and Ω (*ε*_*m*0_ = 0.4) components.

To validate our approach, we select *L* = 10 with fixed modulation depth *a*_*m*_ = 0.77 while varying *ε*_*m*0_, as shown in Figure 3c. Two representative simulation parameters are identified: a circle for fundamental frequency (Ω/2) amplification (*ε*_*m*0_ = 0.47, Δ*a*_m,t_ < 0) and a triangle for second‐harmonic (Ω) amplification (*ε*_*m*0_ = 0.4, Δ*a*_m,t_ > 0). Figure 3d displays the simulated |*E*_*y*_| field distributions. The simulation initializes with a broadband wave packet (center wavelength = 8π*c*/3 Ω, wave packet width = 2π*c*/Ω) at *z* = 5, interacting with the MO‐PTC slab centered at *z* = 15. Although the temporal evolution clearly shows wave amplification, the frequency components cannot be resolved. For precise frequency characterization, we analyze the frequency spectrum of the *E*_*y*_ field at *z* = 25 during *t* = 0–300 via fast Fourier transform [Figure 3e], revealing (see Video S2): For *ε*_*m*0_ = 0.47, the amplified light oscillates at Ω/2 (half the modulation frequency); for *ε*_*m*0_ = 0.4, the amplified light maintains the modulation frequency Ω. This controlled frequency selection, achieved through simple adjustment of the magneto‐optic parameter *ε*_*m*0_, conclusively demonstrates our scheme's capability for active frequency management in finite PTC systems.

## 5 Conclusions

In this Letter, we demonstrate polarization‐ and frequency‐selective light amplification in spatially finite MO‐PTCs through threshold engineering. By applying an external magnetic field perpendicular to the propagation direction, we induce tunable birefringence that modifies the effective permittivity of e‐light (*ε*_*e*_). Crucially, the quadratic dependence of *ε*_*e*_ on the magneto‐optic parameter *ε*_*m*_ generates both fundamental and second‐harmonic modulation components. Furthermore, we identify a feasible material platform that has the potential to implement our proposed concept (see Supporting Information S1). The characteristic oscillatory dependence of amplification thresholds on optical distance enables robust discrimination between different polarization states and frequency components. This work establishes a new paradigm for controlling light in finite time‐varying systems and expands the toolkit for wave manipulation in anisotropic media.

## Author Contributions

**Shuqiao Xu:** writing – original draft, conceptualization, methodology, software, data curation, investigation. **Haoshuang Zhong:** validation. **Zicheng Yang:** formal analysis. **Guoyong Zhang:** supervision. **Hao Chen** **:** visualization. **Xiao Chen:** visualization. **Bo Chu:** supervision. **Zhuo Chen:** writing – review and editing, project administration, resources, funding acquisition. **Zhenlin Wang:** funding acquisition, project administration, resources.

## Conflicts of Interest

The authors declare no conflicts of interest.

## Supporting information


Supporting Information S1

**Video S1:** Temporal evolution of the wave for polarization‐selective amplification in Figure 2(d,e).

**Video S2:** Temporal evolution of the wave for frequency‐selective amplification in Figure 3(d).

## Data Availability

The data that support the findings of this study are available from the corresponding author upon reasonable request.
