# Supplementary material for: Polarization‐ and Frequency‐Selective Light Amplification in Magneto‐Optic Photonic Time Crystals
Source: Nanophotonics. 2026 Apr 14;15(8):e70069. doi: 10.1002/nap2.70069 (PMC13642151; doi:10.1002/nap2.70069)
Supplement: Supplementary file 1 [file NAP2-15-e70069-s002.pdf]

Supplementary Information for "Polarization- and Frequency-Selective Light Amplification in Magneto-Optic Photonic Time Crystals"

Shuqiao Xu, Haoshuang Zhong, Zicheng Yang, Guoyong Zhang, Hao Chen, Xiao Chen, Bo Chu, Zhuo Chen,* and Zhenlin Wang

School of Physics and National Laboratory of Solid State Microstructures, Nanjing University, Nanjing 210093, China

*zchen@nju.edu.cn

Section 1: Light amplification modulation threshold of a PTC slab 2

Section 2: Band structure of MO-PTC under perpendicular magnetization 3

Section 3: Modulation analysis 4

Section 4: A feasible material platform 5

Section 5: Supplementary video 6

Reference 6

Section 1: Light amplification modulation threshold of a PTC slab

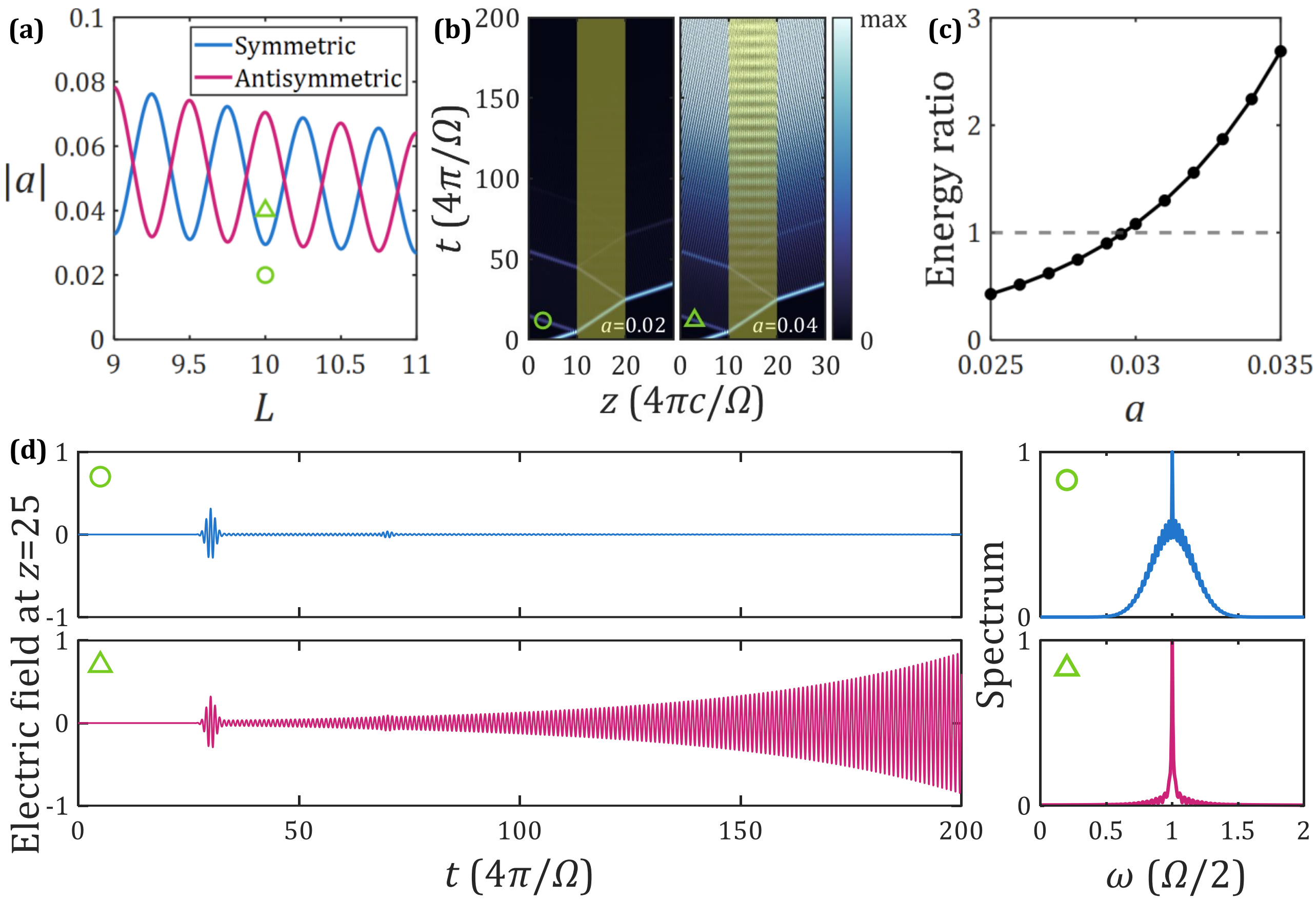

Fig. S1. Light amplification modulation threshold of a PTC slab. (a) Modulation depth to sustain the self-excited oscillations eigenmodes of a PTC slab when n_b=1, with simulation parameters marked for: (i) below threshold (circle, $L = 10$, $a = 0.02$) and (ii) above threshold (triangle, $L = 10$, $a = 0.04$). (b) FDTD simulation results showing electric field $|E|$ evolution. The PTC slab (centered at z = 15) interacts with an initially wave packet (centered at z = 5, center wavelength = $4\pi c/\Omega$, wave packet width = $4\pi c/\Omega$), showing light dissipation (circle) or amplification (triangle). (c) The energy ratio $R = W_2/W_1$ for different modulation depth a and fixed $L = 10$. (d) Electric field evolution at $z = 25 \times 4\pi c/\Omega$ (left) and its fast Fourier transform (right).

Ref. [1] previously analyzed the eigenmodes of a photonic time crystal (PTC) slab under the following modulation scheme:

$$\varepsilon(t) = \bar{\varepsilon}\left[1 + a\cos(\Omega t)\right], \tag{1.1}$$

where we neglect permeability modulation. In finite-thickness slabs, these eigenmodes require a minimum modulation depth to sustain oscillations. Within the small-modulation approximation ($a \ll 1$), the relationship between slab thickness and required modulation depth is given by Eq. (17) in Ref. [1], which we reformulate as:

$$\frac{n_b^2-\bar{\varepsilon}}{n_b^2+\bar{\varepsilon}}\cos\left(2\pi\sqrt{\bar{\varepsilon}}L\right)\pm\cos\left(\frac{\pi\sqrt{\bar{\varepsilon}}La}{2}\right)=0,\quad L=\frac{d\Omega}{4\pi c}. \tag{1.2}$$

Fig. S1(a) displays the characteristic modulation depths supporting steady-state oscillations.

The light amplification gain in PTC slabs increases with modulation depth. When the modulation depth exceeds the critical threshold $|a_{\mathrm{t}}|$, net amplification occurs. To demonstrate this threshold behavior, we perform FDTD simulations (Fig. S1(b)) at $L = 10$, $n_b = 1$, and $\bar{\varepsilon} = 4$ (where $|a_{\mathrm{t}}| = 0.0295$) for two cases:

1) Below threshold (circle, $a = 0.02$): Field dissipation dominates.
2) Above threshold (triangle, $a = 0.04$): Net amplification occurs.

These results validate $|a_{\mathrm{t}}|$ as the critical transition point between dissipative and amplifying regimes. Fig. S1(d) further shows the evolution of the electric field at $z = 25$ (left) and its fast Fourier transform (right), indicating that the amplified light has a frequency of $\Omega/2$.

The threshold is extracted by directly quantifying the net gain or loss in the time-domain simulation. To do this, we compute the transmitted energy flux at $z = 25$. We compare the energy integrated over two adjacent time intervals of equal duration: W_1 (during $t = 100$-150) and W_2 (during $t = 150$-200). The ratio $R = W_2/W_1$ directly measures whether the system's output is growing ($R > 1$) or decaying ($R < 1$) after the initial transient. By simulating a series of modulation depths a for a fixed L, we obtain $R(a)$, as shown in Fig. S1(c). The threshold a_{th} is then precisely determined as the value where $R(a_{\mathrm{th}}) = 1$, obtained via interpolation between data points.

Section 2: Band structure of MO-PTC under perpendicular magnetization

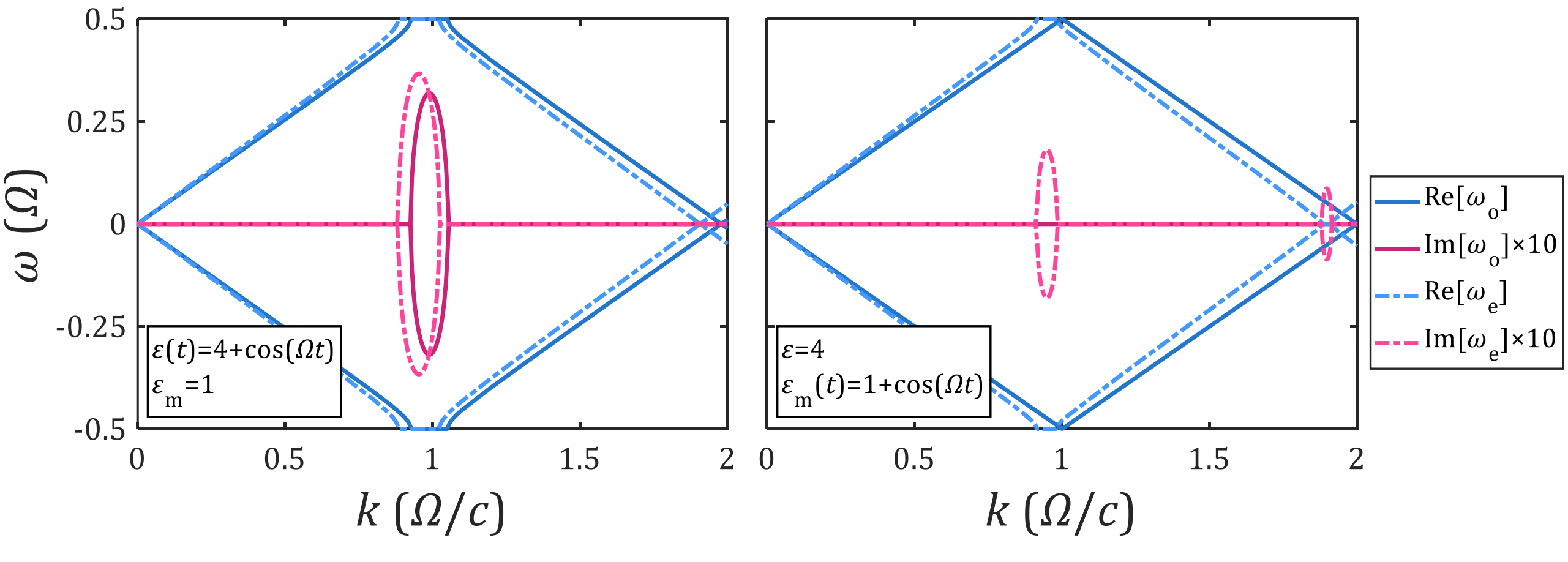

Fig. S2. Band structure analysis of bulk MO-PTC under perpendicular magnetization through the plane wave expansion method. The modulation is applied in ε (left) and ε_{m} (right). Solid and dashed lines represent the frequency band of o-light and e-light, respectively.

In our theoretical framework, we consider light propagation along the z-axis in the presence of an external magnetic field applied in the x-direction. The relative permittivity tensor for the magneto-optic

crystal is given by:

$$\boldsymbol{\varepsilon}=\begin{bmatrix}\varepsilon & 0 & 0\\ 0 & \varepsilon & -i\varepsilon_{\mathrm{m}}\\ 0 & i\varepsilon_{\mathrm{m}} & \varepsilon\end{bmatrix}. \tag{2.1}$$

As derived in Ref. [2] (Section 8.13) and summarized in Table S1, this configuration yields distinct polarization states and effective permittivities for o-light and e-light. Using the plane wave expansion method [3], we calculate the band structures for both polarization states in bulk MO-PTC, shown in Fig. S2. This analysis reveals two key control mechanisms:

1) Polarization-selective amplification: By modulating the diagonal elements (ε), we exploit the different k-gaps for o-light and e-light to achieve polarization-dependent amplification.
2) Frequency-selective amplification: Modulation of the off-diagonal elements (ε_{m}) creates distinct k-gaps at the fundamental frequency and its second harmonic in e-light, enabling frequency-selective control.

	o-light	e-light
$\vec{E}$	$E_x \neq 0,\quad E_y = E_z = 0$	$E_x = 0,\quad i\varepsilon_{\mathrm{m}} E_y = -\varepsilon E_z \neq 0$
$\vec{D}$	$D_x \neq 0,\quad D_y = D_z = 0$	$D_y \neq 0,\quad D_x = D_z = 0$
$\varepsilon_{\mathrm{eff}}$	$\varepsilon_{\mathrm{o}} = \varepsilon$	$\varepsilon_{\mathrm{e}} = \varepsilon - \varepsilon_{\mathrm{m}}^2 / \varepsilon$ (2.2)

Table S1. Electric field, electric displacement field, and effective permittivities of o-light and e-light in magneto-optic crystals in our theoretical framework.

Section 3: Modulation analysis

We now analyze the effective modulation of extraordinary light (e-light) under modulations of the relative permittivity tensor. While ordinary light (o-light) responds directly to diagonal modulations, e-light exhibits more complex behavior.

1) Diagonal elements modulation:

The time-varying diagonal permittivity is given by:

$$\varepsilon(t)=\bar{\varepsilon}\left[1+a\cos(\Omega t)\right]. \tag{3.1}$$

Substituting Eq. (3.1) into Eq. (2.2) yields the e-light response:

$$\begin{aligned}\varepsilon_{\mathrm{e}}(t)&=\bar{\varepsilon}\left[1+a\cos(\Omega t)\right]-\frac{\varepsilon_{\mathrm{m}}^2}{\bar{\varepsilon}}\frac{1}{1+a\cos(\Omega t)}\\&=\bar{\varepsilon}\left[1+a\cos(\Omega t)\right]-\frac{\varepsilon_{\mathrm{m}}^2}{\bar{\varepsilon}}\left[1-a\cos(\Omega t)+a^2\cos^2(\Omega t)\right]+\mathrm{O}\left(a^3\right)\\&=\bar{\varepsilon}_{\mathrm{eff}}\left[1+a_{\mathrm{eff}}^{(1)}\cos(\Omega t)+a_{\mathrm{eff}}^{(2)}\cos(2\Omega t)\right]+\mathrm{O}\left(a^3\right),\end{aligned} \tag{3.2}$$

where the effective parameters are:

$$\overline{\varepsilon}_{\text{eff}} = \frac{2\overline{\varepsilon}^2 - 2\varepsilon_{\text{m}}^2 - a^2\varepsilon_{\text{m}}^2}{2\overline{\varepsilon}}, \quad a_{\text{eff}}^{(1)} = \left(\overline{\varepsilon}^2 + \varepsilon_{\text{m}}^2\right)\frac{a}{\overline{\varepsilon}_{\text{eff}}\overline{\varepsilon}}, \quad a_{\text{eff}}^{(2)} = -\frac{a^2\varepsilon_{\text{m}}^2}{2\overline{\varepsilon}_{\text{eff}}\overline{\varepsilon}}. \tag{3.3}$$

Under small modulation approximation ($a \ll 1$), only the fundamental frequency component remains significant. The e-light amplification threshold, obtained by combining Eq. (3.2) and (1.2), satisfies:

$$\frac{n_b^2 - \overline{\varepsilon}_{\text{eff}}}{n_b^2 + \overline{\varepsilon}_{\text{eff}}}\cos\left(2\pi\sqrt{\overline{\varepsilon}_{\text{eff}}}L\right) \pm \cos\left(\frac{\pi\sqrt{\overline{\varepsilon}_{\text{eff}}}La_{\text{eff}}^{(1)}}{2}\right) = 0, \quad L = \frac{d\Omega}{4\pi c}. \tag{3.4}$$

2) Off-diagonal elements modulation:

For magneto-optic modulation:

$$\varepsilon_{\text{m}}(t) = \varepsilon_{\text{m0}} + a_{\text{m}}\cos(\Omega t). \tag{3.5}$$

Substitution into Eq. (2.2) gives:

$$\begin{aligned}\varepsilon_{\text{e}}(t) &= \varepsilon - \frac{\varepsilon_{\text{m0}}^2 + 2\varepsilon_{\text{m0}}a_{\text{m}}\cos(\Omega t) + a_{\text{m}}^2\cos^2(\Omega t)}{\varepsilon} \\ &= \frac{2\varepsilon^2 - 2\varepsilon_{\text{m0}}^2 - a_{\text{m}}^2}{2\varepsilon} - \frac{2\varepsilon_{\text{m0}}a_{\text{m}}}{\varepsilon}\cos(\Omega t) - \frac{a_{\text{m}}^2}{2\varepsilon}\cos(2\Omega t) \\ &= \overline{\varepsilon}_{\text{eff}}\left[1 + a_{\text{eff}}^{(1)}\cos(\Omega t) + a_{\text{eff}}^{(2)}\cos(2\Omega t)\right],\end{aligned} \tag{3.6}$$

with the effective modulation depth:

$$\overline{\varepsilon}_{\text{eff}} = \frac{2\varepsilon^2 - 2\varepsilon_{\text{m0}}^2 - a_{\text{m}}^2}{2\varepsilon}, \quad a_{\text{eff}}^{(1)} = -\frac{2\varepsilon_{\text{m0}}a_{\text{m}}}{\overline{\varepsilon}_{\text{eff}}\varepsilon}, \quad a_{\text{eff}}^{(2)} = -\frac{a_{\text{m}}^2}{2\overline{\varepsilon}_{\text{eff}}\varepsilon}. \tag{3.7}$$

Notably, the fundamental frequency modulation depends strongly on the DC ε_{m0} component. For second harmonic generation, the threshold condition requires modifying Eq. (1.2) by replacing $\Omega \to 2\Omega$:

$$\frac{n_b^2 - \overline{\varepsilon}_{\text{eff}}}{n_b^2 + \overline{\varepsilon}_{\text{eff}}}\cos\left(4\pi\sqrt{\overline{\varepsilon}_{\text{eff}}}L\right) \pm \cos\left(\pi\sqrt{\overline{\varepsilon}_{\text{eff}}}La_{\text{eff}}^{(2)}\right) = 0, \quad L = \frac{d\Omega}{4\pi c}. \tag{3.8}$$

Section 4: A feasible material platform

As indicated in previous research [4,5], magnetized plasmas are a promising candidate. Their permittivity tensor can be written as [6]

$$\boldsymbol{\varepsilon} = \begin{bmatrix} \varepsilon & -i\varepsilon_{\text{m}} & 0 \\ i\varepsilon_{\text{m}} & \varepsilon & 0 \\ 0 & 0 & \varepsilon_z \end{bmatrix} \tag{4.1}$$

where (under the convention of $e^{-i\omega t}$ time dependence)

$$\varepsilon = 1 - \frac{(\omega_p/\omega)^2 (1 + i\gamma/\omega)}{(1 + i\gamma/\omega)^2 - (\omega_c/\omega)^2}, \quad \varepsilon_{\mathrm{m}} = -\frac{(\omega_p/\omega)^2 (\omega_c/\omega)}{(1 + i\gamma/\omega)^2 - (\omega_c/\omega)^2},$$
$$\varepsilon_z = 1 - \frac{(\omega_p/\omega)^2}{1 + i\gamma/\omega}, \quad \omega_p^2 = \frac{N_e e^2}{m\varepsilon_0}, \quad \omega_c = \frac{|e| B_{\mathrm{ext}}}{m} \tag{4.2}$$

To illustrate the tunability, we plot the tensor components versus the plasma frequency ω_p and the cyclotron frequency ω_c in Fig. R4 (assuming no loss, $\gamma = 0$, and a normalized operating frequency $\omega = 1$). Firstly, by fixing ω_c and varying ω_p (Fig. R4(a)), both ε and ε_z change, implying that both ordinary (o-) and extraordinary (e-) light components can be modulated. This could be used to realize polarization-selective control. Secondly, by fixing ω_p and varying ω_c (Fig. R4(b)), ε_z remains constant while ε and ε_{m} change, meaning that only the e-light component is effectively modulated. This provides a pathway for frequency-selective amplification. Crucially, ω_p and ω_c can be controlled in practice by tuning the electronic density (e.g., via optical pumping or electrical gating) and the external magnetic field, respectively. This independent control over the tensor components provides a direct and realistic material pathway to implement the two types of spatiotemporal modulation (affecting diagonal or off-diagonal elements) required for the selective amplification mechanisms studied in our work.

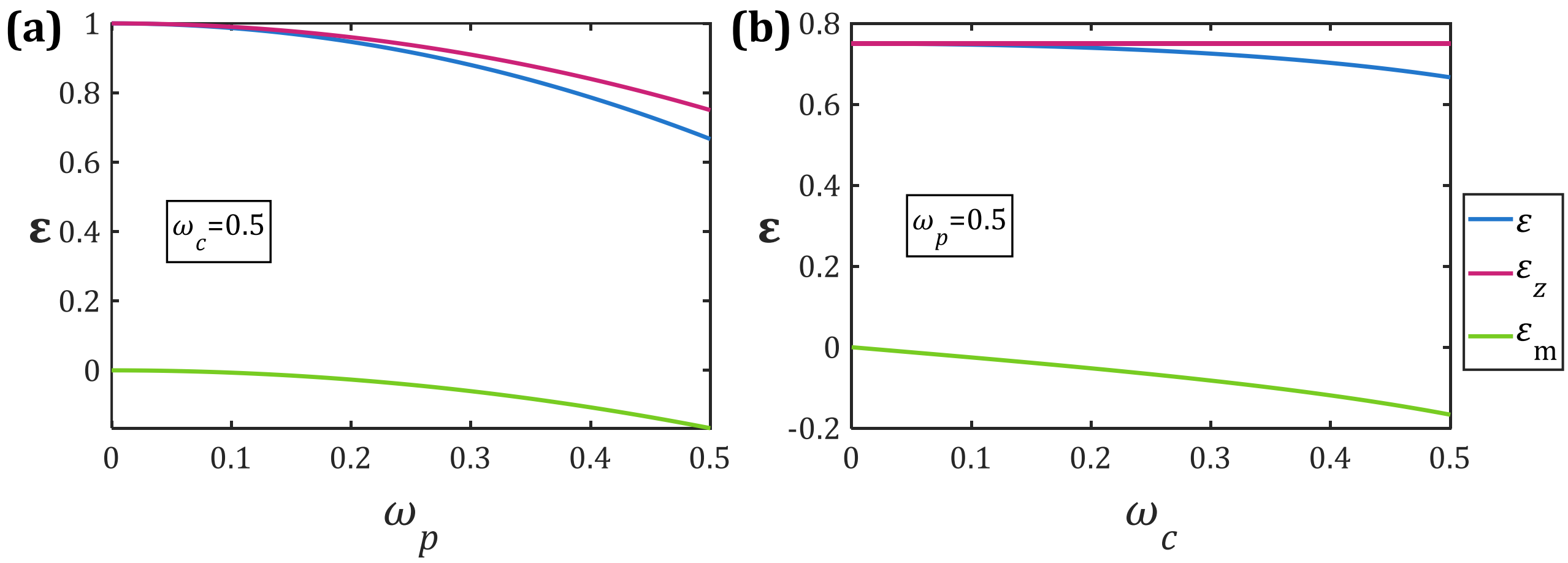

Fig. S3. Permittivity tensor of magnetized plasmas (assuming no loss, $\gamma = 0$, and a normalized operating frequency $\omega = 1$).

Section 5: Supplementary video

Supplementary video 1: Temporal evolution of the wave for polarization-selective amplification in Fig. 2 (d)(e).

Supplementary video 2: Temporal evolution of the wave for frequency-selective amplification in Fig. 3 (d).

Reference

[1] J. S. Martínez-Romero and P. Halevi, Parametric resonances in a temporal photonic crystal slab, Phys.

Rev. A **98**, 053852 (2018).

[2] A. Ishimaru, *Electromagnetic Wave Propagation, Radiation, and Scattering: From Fundamentals to Applications*, 1st ed. (Wiley, 2017).

[3] J. R. Zurita-Sánchez, P. Halevi, and J. C. Cervantes-González, Reflection and transmission of a wave incident on a slab with a time-periodic dielectric function ϵ (t), Phys. Rev. A **79**, 053821 (2009).

[4] H. Li, S. Yin, and A. Alù, Nonreciprocity and Faraday Rotation at Time Interfaces, Phys. Rev. Lett. **128**, 173901 (2022).

[5] H. Yan, Z. Li, X. Li, W. Zhu, P. Avouris, and F. Xia, Infrared Spectroscopy of Tunable Dirac Terahertz Magneto-Plasmons in Graphene, Nano Lett. **12**, 3766 (2012).

[6] A. Ishimaru, *Electromagnetic Wave Propagation, Radiation, and Scattering: From Fundamentals to Applications*, 1st ed. (Wiley, 2017).
